# A Special Additive Enables All Cations and Anions Passivation for Stable Perovskite Solar Cells with Efficiency over 23%

**DOI:** 10.1007/s40820-021-00688-2

**Published:** 2021-08-06

**Authors:** Wenjing Zhao, Jie Xu, Kun He, Yuan Cai, Yu Han, Shaomin Yang, Sheng Zhan, Dapeng Wang, Zhike Liu, Shengzhong Liu

**Affiliations:** 1 Key Laboratory of Applied Surface and Colloid Chemistry, Ministry of Education, Shaanxi Key Laboratory for Advanced Energy Devices, Shaanxi Engineering Lab for Advanced Energy Technology, School of Materials Science & Engineering, Shaanxi Normal University, Xi’an, 710119 People’s Republic of China; 2 Dalian National Laboratory for Clean Energy, iChEM, Dalian Institute of Chemical Physics, Chinese Academy of Sciences, Dalian, 116023 People’s Republic of China

**Keywords:** Additives, Simultaneous passivation, Combined effects, High efficiency, Perovskite solar cells

## Abstract

Passivating undercoordinated ions is an effective way to reduce the defect densities at the surface and grain boundaries (GBs) of perovskite materials for enhanced photovoltaic performance and stability of perovskite solar cells (PSCs). Here, (BBF) complex is chosen as a multifunctional additive, which contains both C_7_H_9_N and BF_3_ groups working as Lewis base and Lewis acid, respectively, can bond with Pb^2+^/I^−^ and FA^+^ on the surface and in the GBs in the perovskite film, affording passivation of both cation and anion defects. The synergistic effect of the C_7_H_9_N and BF_3_ complex slows the crystallization during the perovskite film deposition to improve the crystalline quality, which reduces the trap density and the recombination in the perovskite film to suppress nonradiative recombination loss and minimizes moisture permeation to improve the stability of the perovskite material. Meanwhile, such an additive improves the energy-level alignment between the valence band of the perovskite and the highest occupied molecular orbital of the hole-transporting material, Spiro-OMeTAD. Consequently, our work achieves power conversion efficiency of 23.24%, accompanied by enhanced stability under ambient conditions and light illumination and opens a new avenue for improving the performance of PSCs through the use of a multifunctional complex.

**Supplementary Information:**

The online version contains supplementary material available at 10.1007/s40820-021-00688-2.

## Highlights

- A special additive-(benzylamine)trifluoroboron (BBF) is applied to improve the quality of FAMAPbI_3_ perovskite.
- BBF concurrently passivates cationic and anionic perovskite defects.
- The perovskite solar cell with BBF shows a high power conversion efficiency of 23.24% and an excellent stability.

**Supplementary Information:**

The online version contains supplementary material available at 10.1007/s40820-021-00688-2.

## Graphical abstract

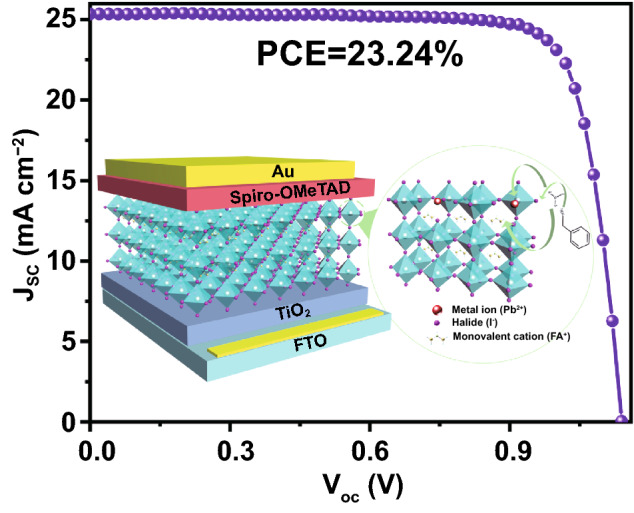

## Introduction

Organic–inorganic hybrid perovskite solar cells (PSCs) have gained enormous attention during the past decade due to their superb photovoltaic properties, low-cost manufacturing technologies, and unprecedented rise in power conversion efficiencies (PCEs) [1, 2]. However, the certified champion PCE (25.5%) of the PSCs is still far from their theoretical value predicted from the Shockley-Queisser (S-Q) theory (~ 31%), mainly due to the open-circuit voltage (*V*_*oc*_*)* loss from nonradiative recombination [3]. Unlike the traditional robust perovskite oxides (e.g., CaTiO_3_), the frail Coulombic interaction and weak ionic bonding cause the vulnerable ions to escape or reorganize in the perovskite film [4, 5], which generates a considerable number of deep defects serving as nonradiative recombination centers and thus results in *V*_*oc*_ and PCE loss in the PSCs. In addition, these deep defects, such as vacancies and undercoordinated ions formed at the surface and GBs of the perovskite, impair the stability of the PSCs [6, 7].

In FAMAPbI_3_ ([FA] formamidinium CH(NH_2_)_2_, [MA] methylammonium CH_3_NH_3_) hybrid perovskite, the larger FA^+^ cation has a smaller dipole moment and a weaker interaction with the [PbI_6_]^2−^ octahedral, which results in the formation of Pb-I antisite defects in at the FA^+^-dominant perovskite surface and causes deep-level defects such as Pb clusters (i.e., Pb^2+^ dimers) [8, 9]. Meanwhile, the volatile component of MA^+^ cations tends to escape from FAMAPbI_3_ perovskite under thermal stress, which results in the formation of undercoordinated Pb^2+^ deep-level defects that serving as electron traps [10]. A great many works have been found that these Pb-related defects (e.g., Pb clusters and uncoordinated Pb^2+^) in FAMAPbI_3_ PSCs lead to deterioration of not only the device PCE but also the stability [11]. Meanwhile, in FAMAPbI_3_ perovskite, due to the large size of the cations, shallow iodine interstitial and vacancy defects can be generated unintentionally during thermal annealing or device measurement processes, which can lower activation energy barrier for transformation from the α phase to δ phase [12].

Recently, various molecules have been developed as additives to heal/reduce FA^+^/MA^+^/I^−^ vacancy or Pb-related defects for suppressing the nonradiative recombination loss in FAMAPbI_3_ PSCs, eventually enhancing the *V*_*oc*_, PCE and stability [13, 14]. However, most of them are supposed to play a single role, and can only passivate one or two charged defects in FAMAPbI_3_ perovskite [15]. There are very few molecules that can passivate both all the FA^+^/MA^+^/Pb^2+^ cations and I^–^ anions in FAMAPbI_3_. Therefore, it is necessary to develop a multifunctional molecule that can passivate all the charged defects in dual-cation perovskite to further enhance the device performance.

Here, we firstly introduced a multifunctional additive (Benzylamine)Trifluoroboron (BBF) that contains both C_7_H_9_N and BF_3_ groups into perovskite precursor because of the following advantages: (1) The new additive (BBF) can retard the crystallization process of the perovskite film to improve the film quality. (2) BBF behaves both as the a Lewis acid (electron acceptor) and the a Lewis base (electron donor) to simultaneously bond with FA^+^/MA^+^/Pb^2+^ cations and I^–^ anions on the surface and at the grain boundaries (GBs) of perovskite films, which can effectively heal/reduce FA^+^/MA^+^ vacancy and Pb-related defects and prevent the movement of I^–^ ions. (3) A surface gradient distribution of BBF can modulate the surface electronic properties of the perovskite, leading to a better match of energy-level alignment between the perovskite and Spiro-OMeTAD. (4) BF_3_ in BBF can increase the hydrophobicity of the perovskite surface to further improve the stability of the PSCs. The combination of the above effects resulted in suppressed nonradiative recombination loss in the PSCs and a significantly increased PCE from 21.60 to 23.24%. Additionally, the device with BBF exhibited a smaller hysteresis and improved ambient and light illumination stability.

## Experimental Section

### Materials

Unless otherwise specified, all of the materials were purchased from Xi’an Polymer Light Technology Corp. (Benzylamine)Trifluoroboron was purchased from Accela ChemBio Co. Ltd. N,N-dimethylformamide (DMF) and dimethylsulfoxide (DMSO) were purchased from Shanghai Aladdin Biochemical Technology Co. Ltd.

### Solar Cell Fabrication

FTO glass was first cleaned by ultrasonic for 30 min with glass cleaner (water to glass cleaner ratio is 100: 1), and then cleaned by ultrasonic for two times with ultra-pure water for 30 min each time. Before deposition of 40 nm TiO_2_ on a clean FTO glass substrate by chemical bath deposition, in accordance with our previously reported method [16], the glass was air-dried and UV treated for 15 min. The 1.0 M FA_0.85_MA_0.15_PbI_3_ precursor solution was prepared by mixing FAI, MAI, PbI_2_ in mixed DMF and DMSO with ratio 4:1. To prepare the FA_0.85_MA_0.15_PbI_3_ film with BBF additive, the different BBF (0.5, 1.0, 1.5, 2.0 mg) was added into 1 mL perovskite precursor solution. Mixture was stirred at room temperature before using. After annealing TiO_2_ on a hot platform at 200 °C for 30 min and UV for 10 min, 50 μL of perovskite precursor solution was spin-coated on the TiO_2_ by two-step anti-solvent spin coating method (spun at 1000 rpm for 10 s and then 4000 rpm for 45 s), and 200 μL of chlorobenzene was dropped at the spin coating for 20 s, followed by thermal annealing at 150 °C for 30 min to obtain dark films. The Spiro-OMeTAD (90 mg mL^−1^) mixed with 4-tert-butylpyridine (36 μL mL^−1^) and 22 μL Li-TFSI (520 mg mL^−1^ in acetonitrile) was spun at 3000 rpm for 30 s to prepare hole-transport layer on perovskite surface to obtain FTO/TiO_2_/perovskite/Spiro-OMeTAD architecture, finally, the gold electrode (~ 80 nm) was thermally deposited on Spiro-OMeTAD, the device active area was defined as 0.09 cm^2^.

### Device Characterization

The *J-V* curves were obtained by a Keithley 2400 source under the AM 1.5 G irradiation (100 mW cm^−2^). All devices were tested from 2 to − 0.1 V at a rate of 10 mV s^−1^. EQE spectra was recorded using a Q Test Station 500TI system (Crowntech, Inc. USA). XRD patterns were taken with D/MAX 2400 diffractometer. XPS and UPS were analyzed using a photoelectron spectrometer (ESCALAB250Xi, Thermo Fisher Scientific), UV/Vis NIR spectrophotometer was used to obtain absorption spectra of perovskite films (Per-kinElmer, Lambda 950), PL and TRPL spectra (excitation at 510 nm) were measured using a FLS980 spectrometer and PicoQuant FluoQuant 300, respectively. Surface and cross-sectional SEM images were perform by a field-emission SEM (HITACHI, SU-8020), AFM images were produced by Veeco Nano Scope IV with a silicon cantilever, FTIR spectra were investigated with a Bruker Vertex 70. For NMR measurements, JNM-ECZ400S/L1 with a frequency of 400 MHz were used, FAI, MAI, PbI_2_, and BBF were dissolve in d6-DMSO. ToF–SIMS characterization was detected by a ION TOF–SIMS 5. Water contact angles were tested using a DataPhysics OCA 20.

## Results and Discussion

### Effect of BBF on Crystallization Process of Perovskite

**Fig. 1:**
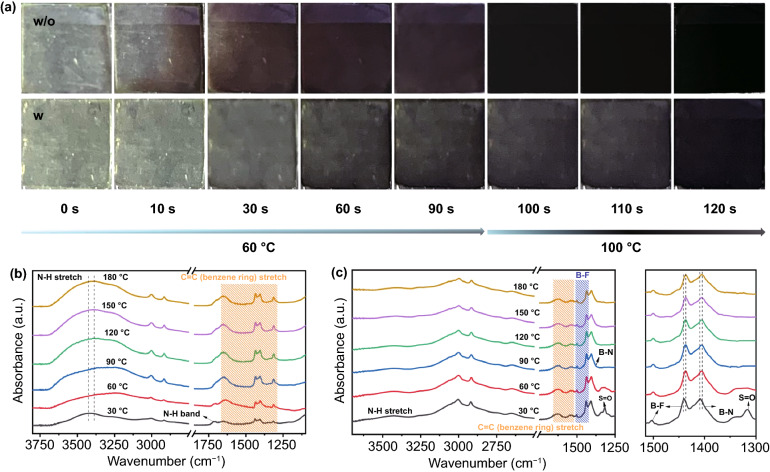
**a** Photographs of perovskite precursor films without and with 1.5 mg mL^−1^ BBF additive annealed at 60 °C/100 °C for different times. FTIR spectra of **b** the FAI precursor film with BBF additive and **c** the PbI_2_ precursor film with BBF additive annealed for 60 s at different temperatures in ambient air (RH: 30%, T: 25 °C)

Figure 1a presents a comparison of photographs of perovskite films with and without BBF additive. Both of the freshly spin-coated films on FTO are transparent, and after annealing for 10 s, the part of the control film begins to turn dark brown, and after 60 s, the whole control film turns dark brown. Compared to the control film, the BBF- modified film obviously takes a long time to finish the same crystallization process, and after annealing for 60 s, the film with BBF shows light gray and needs to be annealed at 100 °C to turn black. For the sake of investigating the role of BBF additive in perovskite growth, the perovskite precursors with and without BBF are annealed at different temperature gradients. As shown in the Fourier transform infrared (FTIR) spectra in Fig. 1b, the stronger absorption peak at 3431 cm^−1^ is attributed to the N–H stretching vibration in FA/MA, and the spectral characteristic between 1200 and 1600 cm^−1^ is assigned to the stretching vibration absorption of C=C in the benzene ring. With the increase of annealing temperature, the N–H stretching vibration shifts to lower wavenumber, and the intensity of the above peaks is strengthened, indicating that there is a strong interaction between the BBF and FAI, which is due to the formation of hydrogen bonds (N–H···F) between the FA/MA and F species. The FTIR spectra of the precursor film containing only BBF and PbI_2_ is also measured and displayed in Fig. 1c. The spectrum exhibits the characteristic peaks of B-N (~ 1380 cm^−1^), B–F (~ 1445 and 1498 cm^−1^), C=C from the benzene ring (~ 1630 cm^−1^), C–H (~ 3005 and 2915 cm^−1^), and N–H (~ 3441 cm^−1^). When the film is annealed at 60 °C for 60 s, the S=O peak from DMSO disappears, and the B–F and B–N bonds move in the direction of lower wavenumber, manifesting that the formation of ionic bonds between F and Pb impaired the interaction between B and F. According to the FTIR results, BBF can simultaneously bond with FA^+^, MA^+^, and Pb^2+^ cations to form some new adducts, such as BBF-FAI, BBF-MAI and BBF-PbI_2_, and the large steric hindrance of these adducts will inhibit the direct reaction between MAI/FAI and PbI_2_ [17]. Therefore, during the Ostwald ripening process of the perovskite precursor film with BBF [18, 19], BBF will reduce the amount of perovskite nucleation, slow the crystallization, and increase the perovskite grain size to improve the film quality. When the film is annealed at 180 °C, B–F and C=C peaks still exist in the FTIR spectrum, which verifies the presence of BBF molecules in the final perovskite film, the thermogravimetric analysis in Fig. S1 and the X-ray photoelectron spectroscopy (XPS) data in Fig. S2 also support this conclusion. The remaining BBF molecules form ionic bonds and hydrogen bonds with the uncoordinated metal and organic cation defects, and these bonds can passivate the defects on the surface and GBs of the perovskite films [20].

### Interaction between BBF and MAI/FAI

**Fig. 2:**
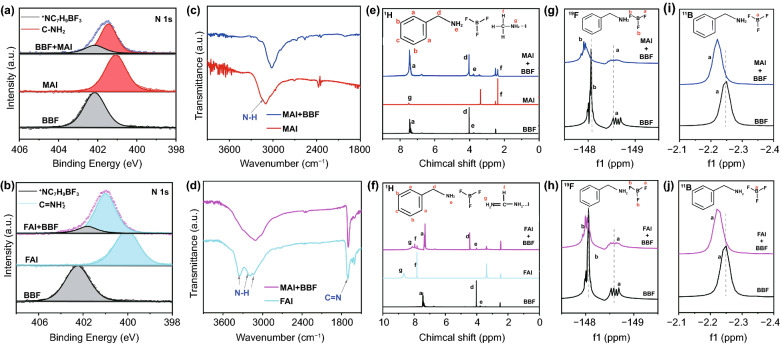
N 1s XPS of **a** BBF, MAI and BBF + MAI and **b** BBF, FAI and BBF + FAI. FTIR of **c** MAI, MAI + BBF and **d** FAI, FAI + BBF. ^1^H NMR of **e** BBF, MAI and BBF + MAI and **f** BBF, FAI and BBF + FAI, ^19^F NMR of **g** BBF, MAI + BBF and **h** BBF, FAI + BBF, ^11^B NMR of **i** BBF, MAI + BBF and **j** BBF, FAI + BBF

In order to further elucidate the interaction between BBF and MAI/FAI in perovskite, a series of characterizations such as XPS, FTIR, and Nuclear magnetic resonance (NMR) are carried out. As shown in Fig. 2a, b, the N 1s signal at 402.15 eV is detected for the pristine BBF film, and the typical bonds of C–N and C=N are detected at 400.09 and 401.08 eV corresponding to MA^+^ and FA^+^ cations, respectively. In the N 1s XPS spectra of the composites (BBF + MAI or BBF + FAI), the feature peaks corresponding to C–N and C=N bonds shrink and shift to higher binding energies compared with pure MAI and FAI, which is due to the formation of hydrogen bonds (N–H···F) between the MAI/FAI and F from BBF. This interaction is also confirmed by the FTIR spectra (Fig. 2c, d), where compared to the pure FA/MA, the N–H vibration in FAI-BBF/MAI-BBF composite shows a shift toward lower wavenumber [21]. The liquid-state ^1^H, ^19^F, and ^11^B NMR measurements also confirm the formation of hydrogen bonds between the F in BBF and MAI/FAI. Figures 2e-h and S2, S3 make clear that the characteristic ^1^H (H_d_ and H_e_ close to N that interact with ^19^F) NMR peaks in BBF show an obvious shift to higher δ value and broadening when mixed with FAI/MAI, which suggests that the interaction between the N and F in BBF is attenuated due to the formation of hydrogen bonds between N in FAI/MAI and F in BBF [22, 23]. The newly formed hydrogen bonds between BBF and FAI/MAI can weaken the shielding of protons on H_d_ /H_e_ in BBF and result in shifting of the resonance to a lower magnetic field, corresponding to higher δ value [24].

### Interaction between BBF and PbI_2_

**Fig. 3:**
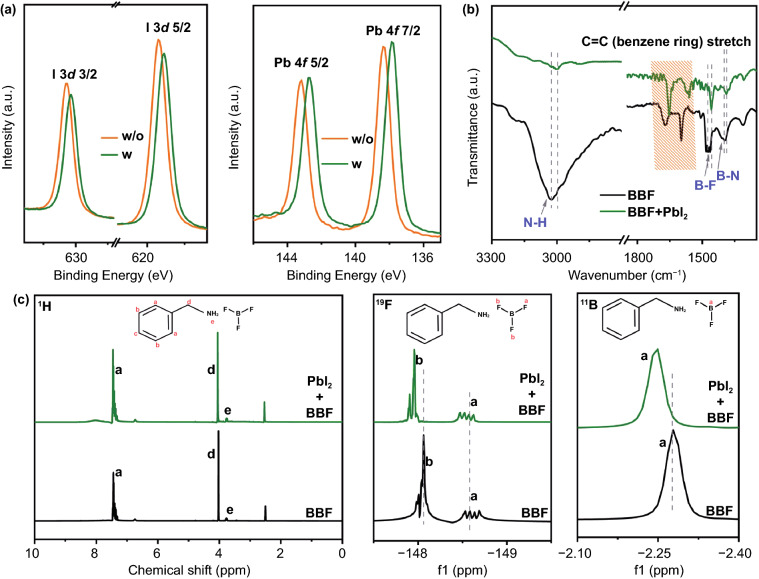
**a** I 3d and Pb 4f XPS spectra of the perovskite film with and without BBF additive. **b** FTIR spectra of BBF solution with and without PbI_2_. **c** ^1^H, ^19^F, ^11^B NMR of BBF solution with and without PbI_2_

For hybrid FAMAPbI_3_ perovskite, uncoordinated metal cations at the crystal surface are unstable in the presence of oxygen and water and will form lead oxide and hydroxide species when exposed to air [25]. Therefore, the passivation on the metal cations in hybrid perovskite is also important. Compared with pure PbI_2_, the binding energies (BEs) of Pb 4f and I 3d XPS spectra are both shifted to lower energy by ~ 0.5 eV in the PbI_2_-BBF composite (Figs. 3a and S2). The BE shift of Pb 4f originates from the ionic bond (Pb–F) interaction between Pb^2+^ and the BBF, meaning that the BBF can passivate the uncoordinated Pb^2+^, thereby reducing the formation of metallic lead. The BE shift of I 3d may be due to the hydrogen bond (N–H···I) between N–H in BBF and iodide in PbI_2_ [26]. Therefore, BBF modification can not only passive the Pb and I defects but also influence the band structure of the perovskite because the Pb 4f and I 3d core levels are closer to the Fermi level of the perovskite [27]. The interaction between PbI_2_ and BBF is further confirmed by FTIR spectra and NMR. As shown in Fig. 3b, compared with pure BBF, the N–H and B–F stretching vibrations in BBF show an obvious shift to lower wavenumber in PbI_2_-BBF composite, providing evidence to confirm the interactions between BBF and PbI_2_ observed in the XPS spectra [28]. Figure 3c shows the ^1^H, ^19^F, and ^11^B NMR patterns of the pure BBF and PbI_2_-BBF composite, respectively. All the characteristic ^1^H, ^19^F, and ^11^B peaks of BFF in PbI_2_-BBF composite show an obvious shift to higher δ value in comparison to pure BBF. The shift of ^1^H is obvious in the magnified spectra in Fig. S5, and this shift is ascribed to the interaction between the N–H bonds in BBF and I ions of PbI_2_ [29]. The shift of ^19^F is due to the interaction between F and Pb, where the valence electron deviates from the proton, and the shielding effect is weakened, so the signal peak appears in the low field. Meanwhile, the interaction force induces a decrease of the electron cloud density around F and an enhancement of the de-shielding effect, which leads to the shift of ^11^B NMR peak to higher δ value. All these interactions between BBF and perovskite precursor will retard the crystallization process of perovskite film, as described in Fig. 1a, which is beneficial to promoting the quality of the perovskite film [30].

### Modification Effect of BBF on Perovskite Film

**Fig. 4:**
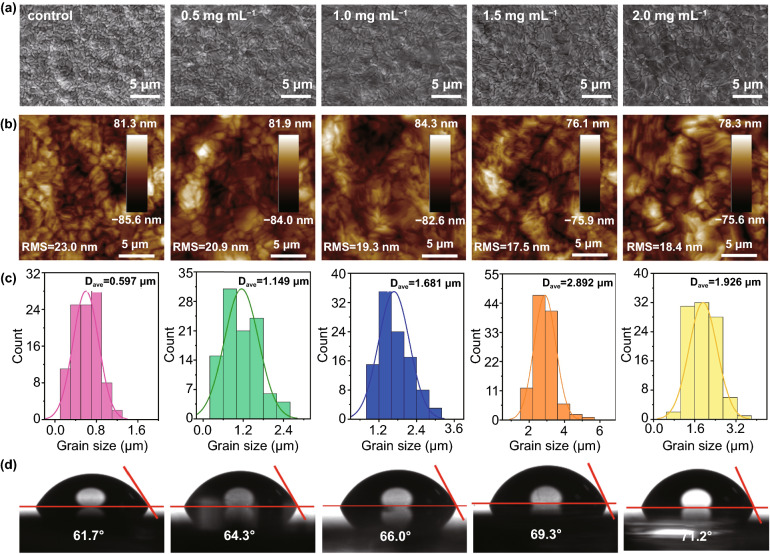
**a** Top-view SEM images. **b** AFM images. **c** grain size distributions. **d** contact angles of perovskite films with different concentrations of BBF additives

Then, the influence of BBF additives on the morphology and crystallinity of perovskite films was characterized by scanning electron microscopy (SEM), atom force microscopy (AFM), and X-ray diffraction (XRD). As shown in Fig. 4a, b, all the perovskite films with and without BBF additives show smooth surfaces, and the average roughness (*R*_*a*_) of the perovskite film with an optimal BBF additive concentration (1.5 mg mL^−1^) is about ~ 17 nm, which is only 5 nm less than that of the control film. However, all the films show different grain sizes, and in order to more intuitively perceive the change of the grain size, a statistical analysis of grain size distribution is obtained from SEM images (Fig. 4c). For the control film, the grain size ranges from 200 to 1290 nm with an average grain size of 597 nm, which is consistent with the result reported by us earlier [31]. The grain size of the perovskite film improves with the increase of BBF additive concentration. When the additive concentration reaches 1.5 mg mL^−1^, the grain size of the perovskite is maximum, with the largest grain of ~ 5.8 μm and average size of 2.89 μm, which is about ~ 6 times larger than that of the control film. However, the grain size decreases when the concentration of additive increases to 2.0 mg mL^−1^, possibly because excessive BBF in the precursor solution affects the Gibbs free energy of nucleation of the perovskite grain [32]. Figure S6 depicts the cross-sectional SEM images of perovskite films without and with optimized BBF additive. The control film exhibits clear grain boundaries and small grain size (∼500 nm), while the perovskite film with BBF additive is an aggregate of compact and large grains and has almost no grain boundaries. The SEM results indicate that BBF can simultaneously affect the crystallinity and density of the perovskite film. As shown in Fig. S7, XRD patterns of the perovskite films with and without BBF are similar and match with the typical black phase perovskite peak, with the diffraction peaks at 14.1°, 28.2°, and 31.6° corresponding to (001), (002), and (012) crystal planes of tetragonal FAMAPbI_3_. When the concentration of BBF additive is 1.5 mg mL^−1^, the intensity of the reflection peak reaches a maximum, while the full width at half maximum (FWHM) of the (001) plane achieves a minimum value. The diffraction peaks of all the perovskite films have no movement, indicating that BBF is not doped into the crystal lattice, but rather exists on the boundary or the surface of the film. Then, the distribution of BBF in perovskite film is detected by time of flight secondary ion mass spectroscopy (ToF–SIMS) (Fig. S8). It is interesting to find that C_7_H_9_N in BBF is mainly distributed on the surface of the perovskite, while BF_3_ in BBF shows an exponential decay in the perovskite film. This special distribution is beneficial to improving the hydrophobicity of the perovskite due to surface enrichment by fluorine and benzene groups, which is confirmed by the increase of deionized water contact angle of the perovskite films with the increase of additive concentration, and this increased hydrophobicity benefits the stability of the perovskite film.

**Fig. 5:**
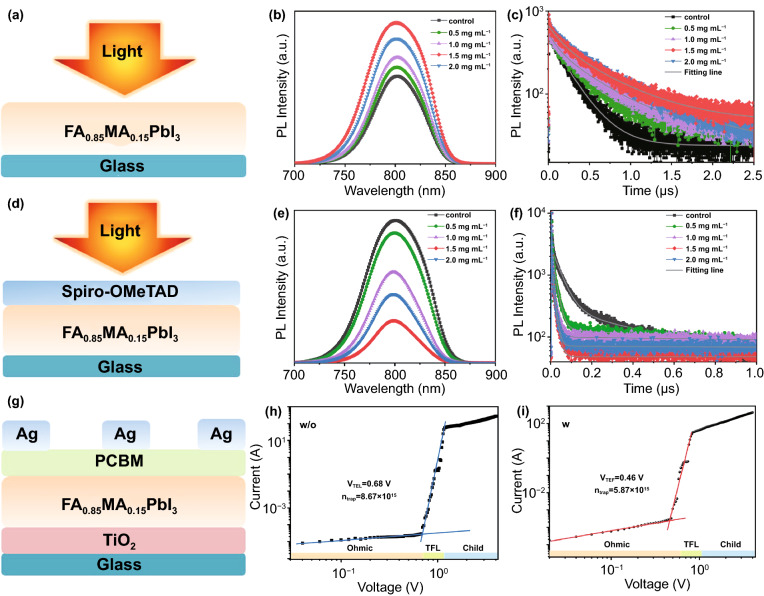
**a** Schematic of film architecture (perovskite/glass) for PL measurements. **b** Steady-state PL spectra and **c** PL decay curves of perovskite films with and without BBF additive. **d** Schematic of film architecture (Spiro-OMeTAD/perovskite/glass) for PL measurements. **e** Steady-state PL spectra and **f** PL decay curves of Spiro-OMeTAD/perovskite/glass. The space-charge-limited current versus voltage of **g** devices (FTO/TiO_2_/perovskite/PCBM/Ag) fabricated **h** without and **i** with BBF additive

To gain insight into the quality of the perovskite films and charge-carrier kinetics, including the effect of BBF concentrations on the photo-excited charge-carrier transport behavior from the perovskite layer to Spiro-OMeTAD, the steady-state PL and time-resolved PL (TRPL) are measured. As shown in Fig. 5a-c, all of the perovskite films exhibit a strong luminescence characteristic peak at ~ 800 nm, which is consistent with the absorption edge of FAMAPbI_3_ perovskite [33]. The perovskite film with optimized BBF additive exhibits the highest emission intensity, which indicates high crystal quality, as confirmed in the SEM and XRD results, and a low nonradiative recombination. The TRPL decay parameters listed in Table S1 are fitted by the bi-exponential decay function f(t) = A_1_exp(− t/τ_1_) + A_2_exp(− t/τ_2_) [34, 35], where A_1_ and A_2_ are the corresponding decay amplitudes, and τ_1_ and τ_2_ are the fast and slow decay times, respectively. As expected, the average carrier lifetime (τ_ave_) has increased by ~ two times from 242.63 to 676.66 ns with optimized BBF additive, confirming that the BBF treatment can decrease the trap-assisted recombination.

Figure 5d-f exhibits the steady-state PL and TRPL decay for the perovskite/Spiro-OMeTAD films. The perovskite films with BBF additive show faster PL quenching and shorter carrier lifetime than the control device. The TRPL curves are also fitted by the bi-exponential decay function f(t) = A_1_exp(− t/τ_1_) + A_2_exp(− t/τ_2_), and the fitted data are listed in Table S2. τ_1_ and τ_2_ are dominated by carrier transfer at the perovskite/Spiro-OMeTAD interface and radiative recombination of trapped charges from the bulk perovskite, respectively [36]. The fitted data establish that the BBF additives can both improve carrier transfer at the interface and reduce nonradiative recombination in the perovskite, which may be due to the gradient distribution of BBF that can adjust the surface band structure of the perovskite [37, 38].

The space-charge-limited current (SCLC) technique is conducted to quantitatively estimate the defect density in the perovskite using a device structure of FTO/TiO_2_/perovskite/PCBM/Ag (Fig. 5i-g). The trap-filled limit voltage (V_TFL_) and calculated trap density of the perovskite film with BBF additive are 0.45 V and 5.87 × 10^15^ cm^−3^, respectively, which are lower than those of the control film (0.68 V and 8.67 × 10^15^ cm^−3^). The reduced trap density is accredited to the high quality of the perovskite film with good passivation and high crystallinity.

### Performance of Devices with BBF Additive

**Fig. 6:**
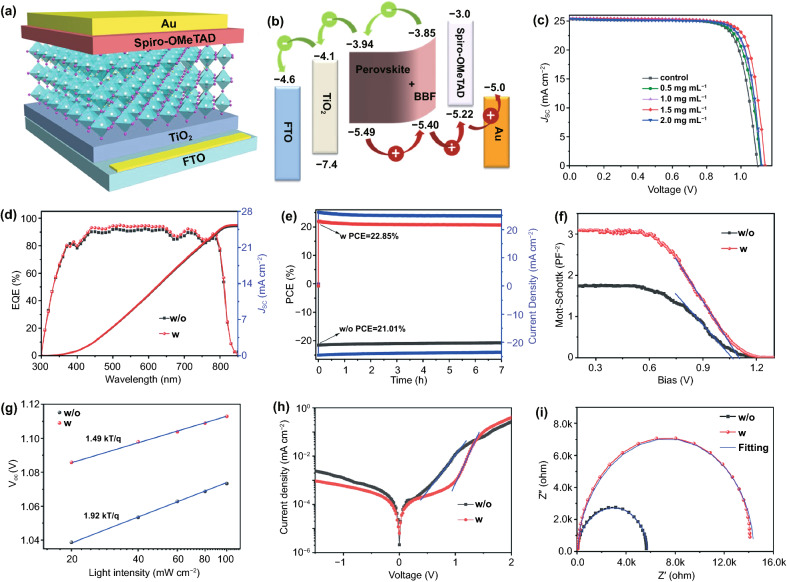
**a** Schematic image of the hybrid PSC device with the structure FTO/TiO_2_/perovskite/Spiro-OMeTAD/Au. **b** Schematic energy-level alignment of the hybrid PSCs with BBF additive. **c** *J-V* curves of the hybrid PSCs with different concentrations of BBF additive. **d** EQE spectra. **e** stable output. **f** Mott-Schottky plots. **g** *V*_*OC*_ dependence on light intensity. **h** dark current–voltage characteristic and **i** Nyquist plots of the hybrid PSCs with and without BBF additive

To investigate the influence of BBF additive on the photovoltaic performance of PSCs, a typical planar-structured PSC is fabricated (Fig. 6a). Ultraviolet photoemission spectroscopy (UPS) is conducted to explore the effect of BBF additive on the energy band characters of the perovskite films. The band gap of the perovskite film is almost unchanged after the addition of BBF, as confirmed by the absorption (Fig. S9) and PL emission (Fig. 5b) spectra. As shown in Figs. 6b and S10, the BBF additive causes both the valence band maximum and conduction band minimum of the perovskite to move up, which can reduce the energy barrier and loss for hole transfer from the perovskite to Spiro-OMeTAD while blocking the electrons from moving to the Spiro-OMeTAD for reducing the interface recombination [39].

Figure 6c depicts the *J-V* characteristics of the planar PSCs with different concentrations of BBF additives, and Table 1 exhibits their photovoltaic parameters. It is quite obvious that the PSC with optimized BBF additive (1.5 mg mL^−1^) delivers a champion photovoltaic performance with a short-circuit current density (*J*_*sc*_) of 25.33 mA cm^−2^, an open-circuit voltage (*V*_*oc*_) of 1.14 V, and a fill-factor (FF) of 0.81, achieving a PCE up to 23.24%. In contrast, the control PSC has a relatively inferior performance with a *J*_*sc*_ of 25.35 mA cm^−2^, a *V*_*oc*_ of 1.10, and a FF of 0.77, resulting in a PCE of 21.6%. According to the photovoltaic data, the *V*_*oc*_ and FF increase obviously, which is attributed to a suitable band alignment between the perovskite with BBF and the Spiro-OMeTAD. *J*_*sc*_ remains almost unchanged with the addition of BBF, and the values are confirmed by the EQE spectra (Fig. 6d). The integrated *J*_*sc*_ values of the devices with and without BBF additive are 25.33 and 25.35 mA cm^−2^, respectively, which match well with the values obtained from the *J-V* curves in Fig. 6c. As shown in Fig. S11 and Table S3, the device with BBF additive displays similar *J-V* curves under forward and backward scanning, showing a small hysteresis index (HI) of 2.4%, whereas the HI for the control device is 12.1%. The much smaller hysteresis factor for the optimized device is attributed to the efficient charge extraction and transport at the perovskite interface. The steady-state current densities at the maximum power points of the PSCs are measured for 7 h (Fig. 6e). The stabilized PCE for the control device is 21.01%, whereas that of the device with BBF additive is 22.85%. To determine the reproducibility of the PSCs, 30 distinct devices are fabricated using the same procedure, with the statistical photovoltaic parameters presented in Fig. S12. All the key parameters exhibit fairly narrow distributions, indicating that all the devices have good reproducibility.

**Table 1:** Summary of the photovoltaic parameters of the hybrid PSCs with different concentrations of BBF additives

Devices (mg mL^−1^)	*V*_*OC*_ (V)	*J*_*SC*_ (mA cm^−2^)	FF	PCE (%)
control	1.10	25.35	0.77	21.60
0.5	1.11	25.33	0.78	22.03
1.0	1.12	25.38	0.79	22.32
1.5	1.14	25.33	0.81	23.24
2.0	1.12	25.36	0.80	22.65

The surface-accumulated BBF can change the band structure of the underlying perovskite film, which will influence the built-in potential (*V*_*bi*_) of the PSCs [30]. The *V*_*bi*_ of devices is assessed via a capacitance versus voltage (Mott-Schottky) analysis. As shown in Fig. 6f, the PSC with BBF additive displays a higher *V*_*bi*_ (1.11 V) than the control PSC (1.07 V). The improved *V*_*bi*_ benefits the *V*_*oc*_ of PSCs and assists in charge transport and collection. To gain insight into the recombination kinetics within PSCs, the *V*_*oc*_ versus light intensity of the device is measured and presented in Fig. 6g. According to the literature [40], when the slope of the line strays from 1 kT q^−1^, it reveals trap-assisted recombination. The slope of the line decreases from 1.92 to 1.49 kT q^−1^ with the addition of BBF, which suggests a more effective suppression of trap-assisted charge recombination in the optimized device and is consistent with the TRPL and SCLC results. The dark *J-V* curves of PSCs are also measured and shown in Fig. 6h. By fitting the curves with an equivalent circuit of a Shockley diode [41], it is found that the device with BBF additive possesses better diode behavior, showing relatively smaller saturation current (9.49 × 10^–10^ mA cm^−2^) than the control device (2.60 × 10^–9^ mA cm^−2^), indicating suppressed charge recombination with a higher rectification ratio and higher *V*_*oc*_ in the device with BBF additive [42].

Electrochemical impedance spectroscopy (EIS) is conducted to further elucidate the positive effect of BBF additive on the charge transport properties of the PSCs. Nyquist plots of devices without and with BBF additive are shown in Fig. 6i, and the fitted results are listed in Table S4. The device with BBF additive exhibits lower series resistance (*R*_*s*_) and higher recombination resistance (*R*_*rec*_) compared with the control device, indicating optimized charge transport and suppressed charge recombination [43, 44].

### Effect of BBF on the Stability of Device

**Fig. 7:**
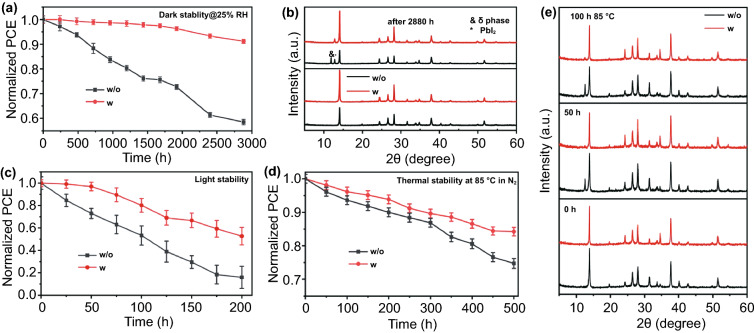
**a** Air stability (humidity: ≈25%). **b** XRD pattern of the hybrid PSCs with and without BBF additive after aging in air for 2880 h. **c** Light stability and **d** thermal stability (T: ≈85 °C in N_2_ atmosphere) of the PSCs with and without BBF additive. **e** XRD patterns of the perovskite films with and without BBF additive after aging at 85 °C in N_2_ atmosphere for 50 h and 100 h

Besides the high photovoltaic parameters, stability is another important metric for PSCs. Therefore, the air stability of perovskite devices is examined. As shown in Fig. 7a, when the unencapsulated PSCs without and with BBF additive are stored in air under relative humidity of ≈25% at 25 °C for 2880 h, the BBF-treated device demonstrates superior stability, maintaining 91% of its initial efficiency, whereas the PCEs of the control PSCs devices drop to 58% of their initial values during the same period. After aging, the perovskite layers were characterized by XRD measurement, and, as shown in Fig. 7b, the δ-phase and PbI_2_ can be observed in the control perovskite film, which means that the control perovskite not only partially changes from α-phase to δ-phase, but also partially decomposed into PbI_2_ and MAI. The peak intensity ratio of the PbI_2_ to (100) diffraction peak of the perovskite is 0.33, which is much higher than that of the perovskite film with BBF additive (0.13). Meanwhile, there is no δ-phase in the aged perovskite film with BBF additive. Therefore, the BBF can effectively enhance the stability of the perovskite film through reducing the phase transition and decomposition, which can be ascribed to the passivation effect and hydrophobicity of BBF, which could effectively reduce the defect density and water/oxygen adsorption and thus stabilize the perovskite lattice.

Additionally, the effect of BBF additive on the light and thermal stabilities of PSCs is also evaluated. As shown in Fig. 7c, when the devices are illuminated under one-sun equivalent intensity for 200 h, the PCE of the control PSC decreases to 16% of its initial value, while the device with BBF additive retains 53% of its initial value. For testing thermal stability, the devices are annealed at 85 °C in a N_2_-filled glove box, and the PCE of the devices with BBF remains at 84% of its initial value for 500 h, whereas the PCE of the control device decreases to 74% of its initial value during the same period (Fig. 7d). Then, the perovskite layers that aged for 50 and 100 h are measured by XRD. As shown in Fig. 7e, it is noted that the perovskite film with BBF additive shows much lower PbI_2_ diffraction peaks and higher perovskite diffraction peaks than those of the control film after 100 h of thermal annealing. The improvement of thermal stability is ascribed to heavy hydrogen bonding between the BBF and perovskite, which reduces the release or decomposition of cations in the perovskite under thermal stress.

## Conclusions

In summary, we demonstrate a facile way to effectively passivate perovskite film by using a multifunctional molecule (BBF) containing both C_7_H_9_N and BF_3_ groups as additive. It is interesting to find that C_7_H_9_N mainly distributes on the surface of the perovskite, while BF_3_ shows an exponential decay in the perovskite film. This special distribution of BBF, which has different coordination ability with perovskite cations and anions, serves multiple functions: it can retard the crystallization process leading to high-quality perovskite film, associate with undercoordinated cations and anions for defect passivation, adjust the surface electronic properties of perovskite forming an energy cascade layer to promote charge transport, as well as increase hydrophobicity of the perovskite film to improve the ambient stability of the perovskite. Relying on the synergistic benefits of the above functions, the BBF-incorporated devices based on the FAMAPbI_3_ absorber deliver a high PCE of 23.24% and high ambient and light illumination stability. The new multifunctional additive (BBF) has the potential to be widely used in low-band-gap perovskites for more efficient perovskite devices.

## Supplementary Information

Below is the link to the electronic supplementary material.

Supplementary file1 (PDF 1137 KB)
