# Supplementary material for: A Special Additive Enables All Cations and Anions Passivation for Stable Perovskite Solar Cells with Efficiency over 23%
Source: Nanomicro Lett. 2021 Aug 6;13:169. doi: 10.1007/s40820-021-00688-2 (PMC8346611; doi:10.1007/s40820-021-00688-2)
Supplement: Supplementary file 1 — Supplementary file1 (PDF 1137 KB) [file 40820_2021_688_MOESM1_ESM.pdf]

Supporting Information for

A Special Additive Enables All Cations and Anions Passivation for Stable Perovskite Solar Cells with Efficiency over 23%

Wenjing Zhao[1], Jie Xu[1], Kun He[1], Yuan Cai[1], Yu Han[1], Shaomin Yang[1], Sheng Zhan[1], Dapeng Wang[1], Zhike Liu[1,]*, and Shengzhong (Frank) Liu[1, 2,]*

[1]Key Laboratory of Applied Surface and Colloid Chemistry, Ministry of Education; Shaanxi Key Laboratory for Advanced Energy Devices; Shaanxi Engineering Lab for Advanced Energy Technology; School of Materials Science & Engineering, Shaanxi Normal University, Xi'an, 710119, P. R. China

[2]Dalian National Laboratory for Clean Energy; iChEM, Dalian Institute of Chemical Physics, Chinese Academy of Sciences, Dalian, 116023, P. R. China.

*Corresponding authors. E-mail: zhike2015@snnu.edu.cn (Zhike Liu); szliu@dicp.ac.cn (Shengzhong (Frank) Liu)

Supplementary Figures and Tables

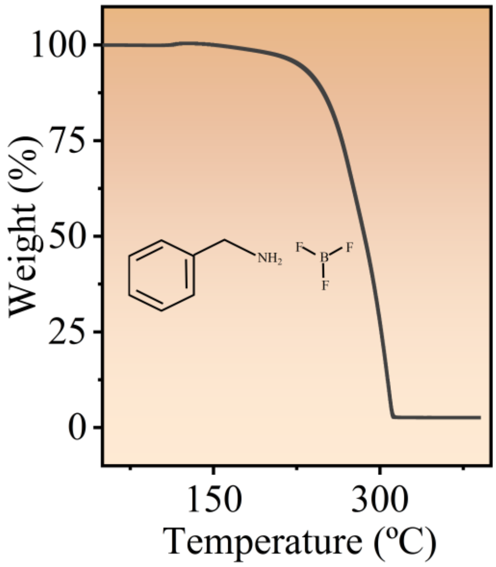

Fig. S1 Thermogravimetric profile for BBF

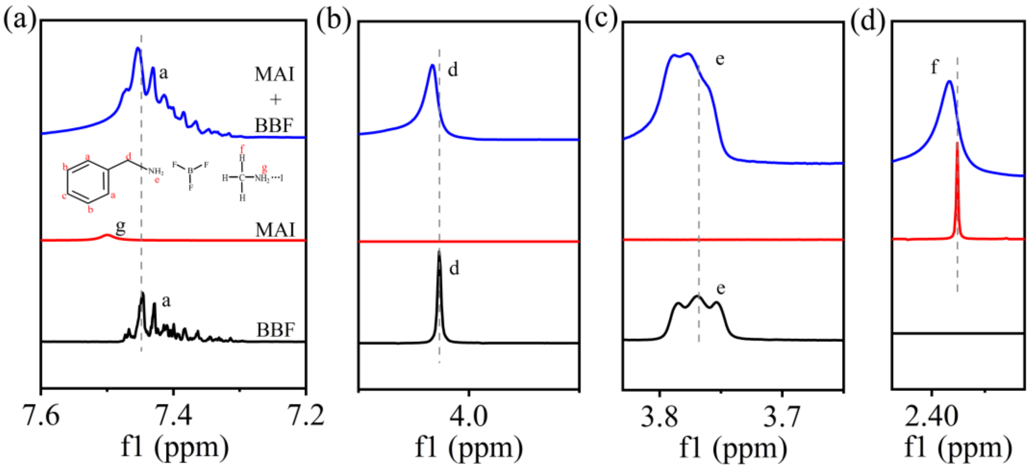

Fig. S2 ^{1}H NMR spectra of BBF, MAI, MAI+BBF

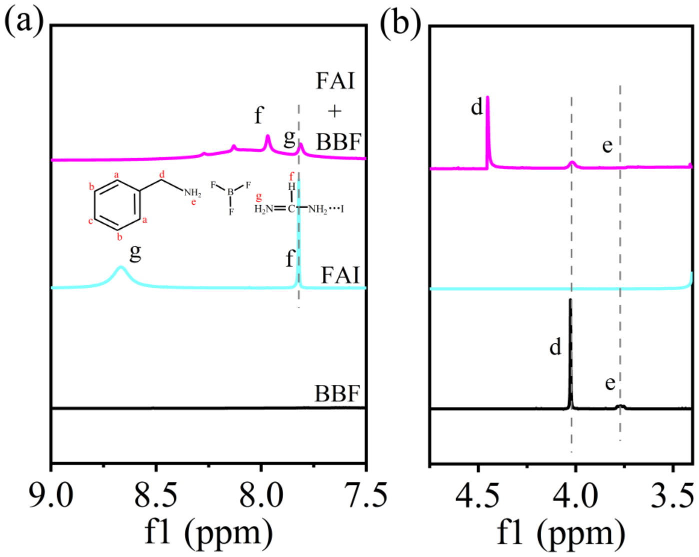

Fig. S3 ^{1}H NMR spectra of BBF, FAI, FAI+BBF

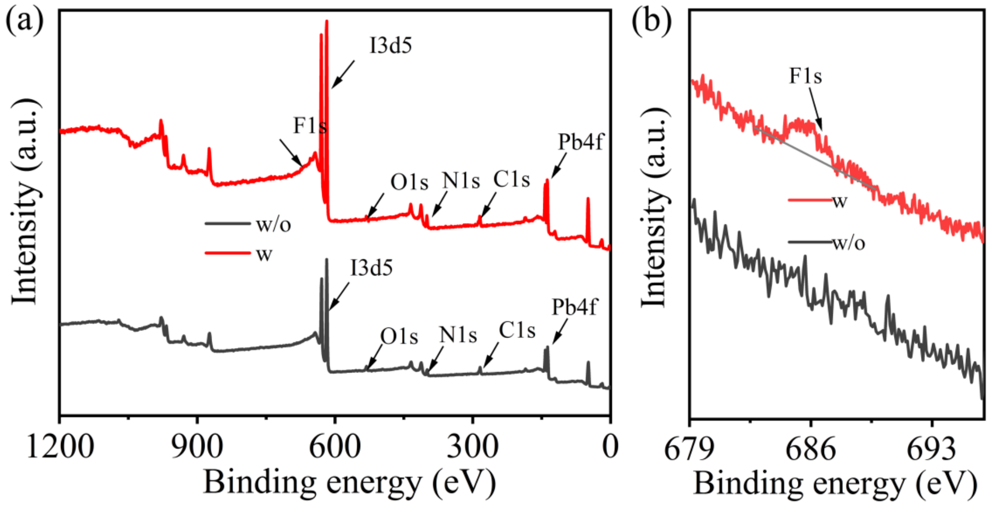

Fig. S4 a Full XPS spectra and **b** F 1s of the perovskite film without and with BBF additive

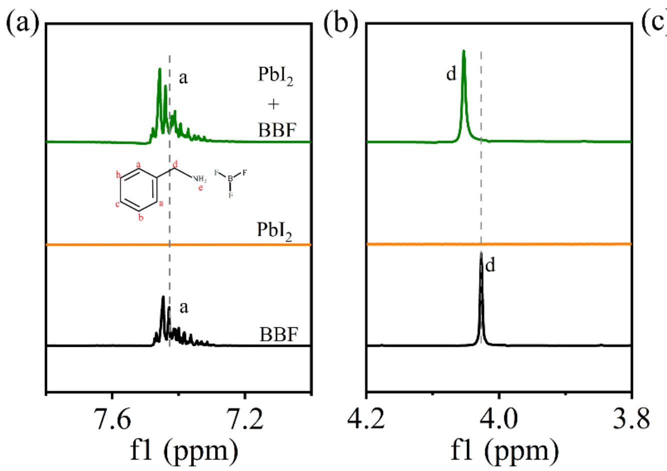

Fig. S5 ^{1}H NMR spectra of BBF, PbI_2, PbI_2+BBF

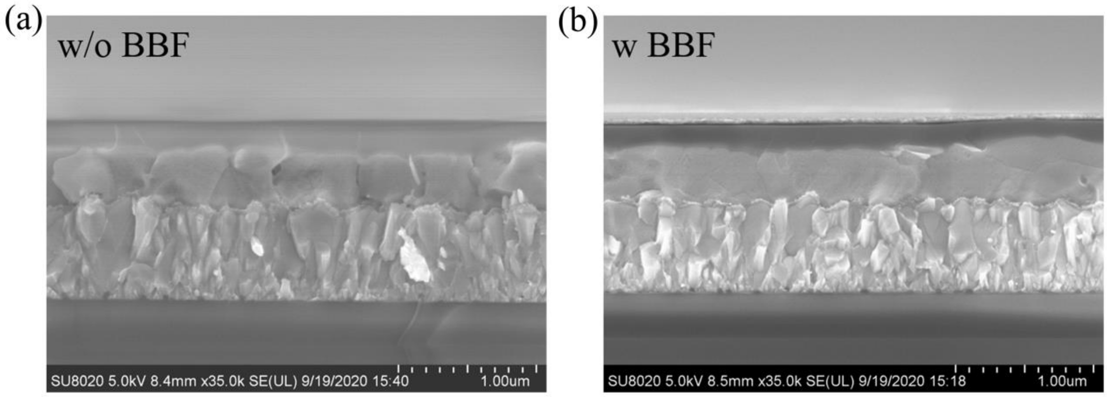

Fig. S6 Cross-sectional SEM images of the PSCs without and with BBF additive

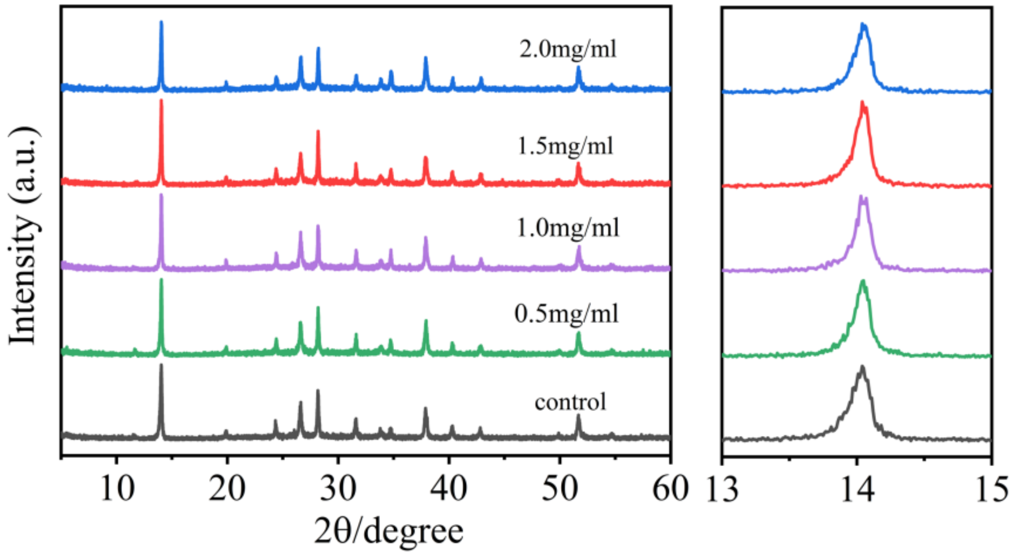

Fig. S7 XRD pattern and (110) diffraction peaks of the perovskite films with different concentrations of BBF additives

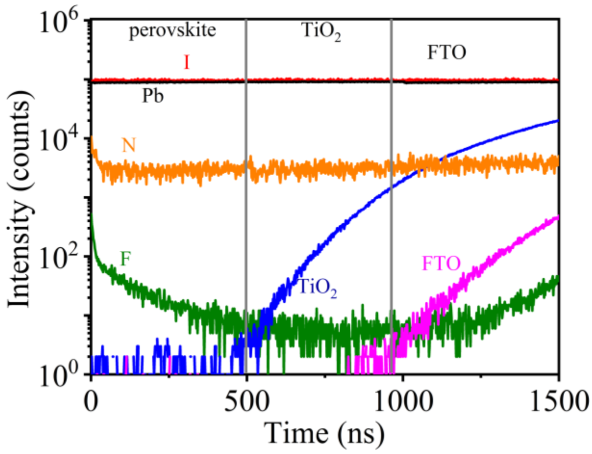

Fig. S8 ToF-SIMS depth profile of the BBF-perovskite film

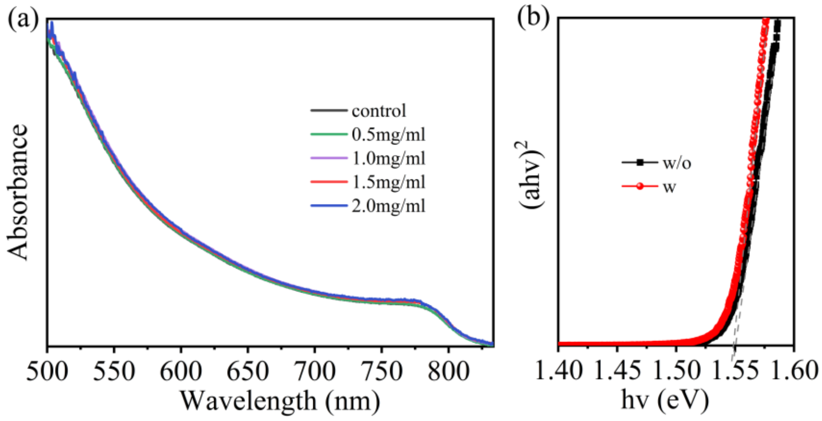

Fig. S9 a UV-Vis absorption spectra and **b** Tauc plots of the perovskite films without and with 1.5 mg/ml BBF additive

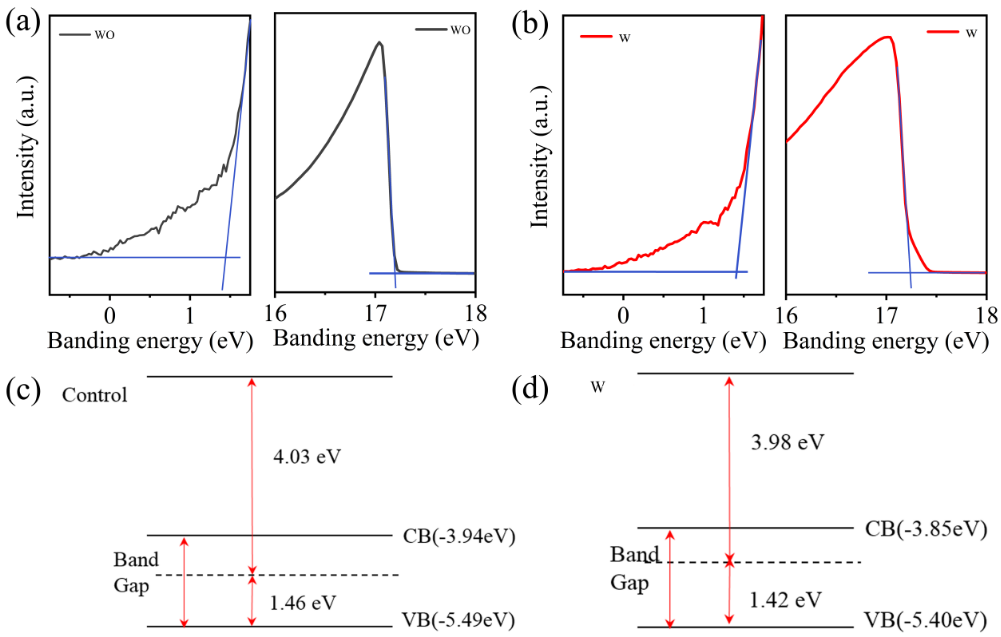

Fig. S10 a, **b** Valence band edges and cutoff regions of UPS spectra and **c**, **d** energy level diagrams for the perovskite films without and with BBF additive

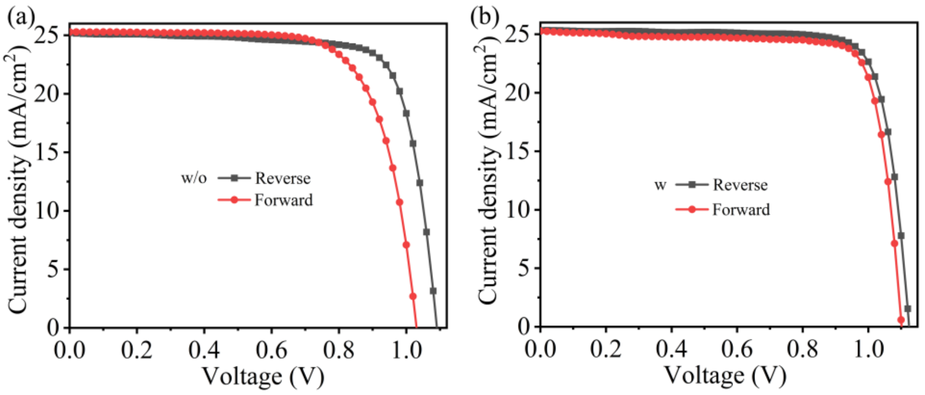

Fig. S11 Reverse and forward *J-V* curves of the PSCs **a** without and **b** with BBF additive

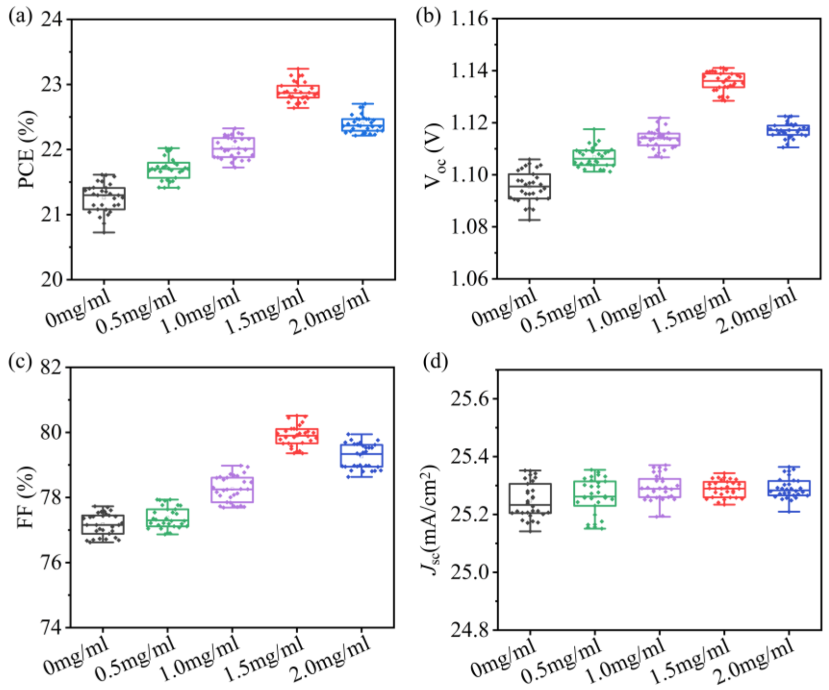

Fig. S12 Box charts of **a** PCE, **b** V_{OC}, **c** FF and **d** J_{SC} of the PSCs (30 samples) with different concentrations of BBF additives

Table S1 Fitting parameters of the TRPL spectra for perovskite films with different concentrations of BBF additives

BBF (mg/mL)	τ_{ave} (ns)	τ_1 (ns)	A_1 (%)	τ_2 (ns)	A_2 (%)
0	242.63	248.25	96.98	62.42	3.02
0.5	401.33	464.05	78.04	178.83	21.96
1.0	521.72	569.74	89.32	120.76	10.68
1.5	676.66	794.73	82.16	132.34	17.84
2.0	634.75	712.16	84.91	199.71	15.09

Table S2 Fitting parameters of the TRPL spectra for Glass/perovskite/Spiro-OMeTAD devices with different concentrations of BBF additives

BBF (mg/ml)	τ_{ave} (ns)	τ_1 (ns)	A_1 (%)	τ_2 (ns)	A_2 (%)
0	184.61	325.02	52.85	27.21	47.15
0.5	179.47	305.51	54.28	29.83	45.72
1.0	163.84	309.61	49.61	20.19	50.39
1.5	97.15	148.92	43.81	5.68	56.19
2.0	142.32	248.96	50.80	32.2	49.20

Table S3 Photovoltaic parameters of the PSCs without and with 1.5mg/mL BBF additive scan in different directions

Device	Direction	V_{OC} (V)	J_{SC} (mA/cm^2)	FF	PCE (%)	Hysteresis Index (%)
w/o	Reverse	1.09	25.21	0.77	21.21	12.1
	Forward	1.03	25.20	0.72	18.65	
w	Reverse	1.12	25.32	0.81	22.97	2.4
	Forward	1.10	25.26	0.81	22.41	

Table S4 EIS fitting parameters for the PSCs without and with BBF additive

Device	R_s (Ω)	R_{tr} (kΩ)	R_{rec} (kΩ)	C_{rec} (F)	C_{tr} (F)	τ_{rec} (µs)
w/o	50.43	0.58	5.10	3.38×10^{-8}	1.21×10^{-8}	17.24
w	36.45	0.36	16.16	7.53×10^{-8}	9.75×10^{-9}	121.62
